# Firmicutes Dysbiosis After Chlorhexidine Prophylaxis in Healthy Patients Submitted to Impacted Lower Third Molar Extraction

**DOI:** 10.3389/fcimb.2021.702014

**Published:** 2021-08-13

**Authors:** Carlos Augusto das Neves, Carlos Henrique Alves, Natália Conceição Rocha, Karina Ferreira Rizzardi, Karolyne Larissa Russi, Alexandre Augusto Albigiante Palazzi, Thaís Manzano Parisotto, Raquel Girardello

**Affiliations:** Laboratório de Microbiologia Molecular e Clínica, Universidade São Francisco, Bragança Paulista, Brazil

**Keywords:** personalized odontology, antibiotic prophylaxis, antibiotic resistance, oral microbiota, oral bacteria and fungi

## Abstract

Prophylaxis with antiseptic and antibiotic therapy is common in impacted lower third molar surgeries, despite the lack of consensus among professionals and researchers in the indication for healthy patients. The aim of the present preliminary study was to verify the impact of prophylaxis therapy with antiseptic and antibiotic in healthy patients submitted to impacted lower third molar extraction, according to oral microorganism quantification. Eleven patients submitted to impacted lower third molar extraction, under prophylactic therapy with 0.12% chlorhexidine and amoxicillin in four experimental phases, were evaluated. Our results showed no significant reduction in total bacteria load, as well as in Bacteroidetes and *C. albicans* loads in the oral cavity, after prophylactic therapy with antiseptic and antibiotic. On the other hand, there was a significant difference between the Firmicutes levels across the follow-up, and this effect seems to be large (*ηp²=0.94*). *Post-hoc* test demonstrated that the levels of Firmicutes in T1 were higher than T0, T2, and T3, suggesting a microbiota dysbiosis, when 0.12% chlorhexidine use, which may be responsible for selection of antibiotic-resistant microorganisms. Our results alert for an overuse of antiseptic and antibiotics by dentists and for a better evaluation of the available protocols.

## Introduction

Third molar extraction is a routine protocol in odontology and diverse techniques for impacted lower third molars are described in the clinical literature, with different degrees of tissue invasion (Kirk et al., 2007; Coulthard et al., 2014). According to Silvestri and Singh (2003), around 65% of healthy individuals have impacted third molars, poorly positioned or with difficulty in hygiene access, with reduced function and a high rate of associated diseases.

Prophylactic therapy using antibiotics and antiseptics are common; however, some authors agree that, to establish a prophylactic treatment with the use of antibiotics, the risk of infection must be significant, regarding the degree of severity, a fact that is not common in extraction of impacted third molars (Lindeboom, 2008; Neves et al., 2020).

There is no consensus among professionals and researchers about the need and the protocol of this therapy in patients without previous infection (Stein et al., 2018; Menon et al., 2019). Microbiological studies are scarce to determine the real role of prophylactic therapy with antimicrobials in healthy patients. Current knowledge around this theme is based mainly in observational studies and systematic reviews (Lodi et al., 2021; Riba-Terés et al., 2021; Yoshida et al., 2021). The aim of this study was to verify the impact of prophylaxis therapy with antiseptic and antibiotic in healthy patients submitted to impacted lower third molar extraction, according to oral microorganism quantification.

## Material and Methods

### Patient’s Records Evaluation

Before the selection of patients for the study, a characterization of the patient’s profile attending a private dental office in Bragança Paulista-SP/Brazil, from 2016 to 2020, was performed. This way, the rate of infections before the surgery procedures, was estimated by analyzing patient’s records.

### Oral Microorganism Quantification

Eleven healthy patients seeking for noninfected impacted lower third molar surgery were selected for oral bacteria quantification by using qPCR. The samples were collected in the same pattern, using a sterile swab (E-swab, Copan), in four different experimental phases: T0 (before surgery), T1 (after 0.12% chlorhexidine mouthwash, for 3 days), T2 (after amoxicillin 875 mg, single dose, 1 h before surgery, followed by amoxicillin 875 mg every 12 h, for 7 days after surgery), and T3 (28 days after amoxicillin use). The DNA was extracted by using Microbiome DNA kit (ZymoBiomics, CA, USA), according to manufacture recommendation. The DNA concentration from clinical samples was normalized and submitted to qPCR in a 7300 Real-Time System (Applied Biosystems, Foster City, CA, USA) for total bacteria, Firmicutes phylum, Bacteroidetes phylum, and *Candida albicans* yeast, according to a protocol previously described (Rizzardi et al., 2021). For each sample, 10 ml of the reaction mixture, including 5 μl of SYBR Green Power up (Thermo Fisher Scientific, Carlsbad, CA, USA), 2.9 μl of ultrapure water, 1.5 μl of DNA sample, and 0.3 μl of each primer were used. Amplification and detection were performed according to the following cycles: 2 min at 50°C, 10 min at 95°C, 40 cycles of 15 s at 95°C, and 1 min at 60°C (Guo et al., 2008).

Total bacterial DNA extracted from the oral cavity was quantified, based on the 466-bp fragment of conservative sequence of *16S rRNA* gene, using specific primers (F: 5′-TCCTACGGGAGGCAGCAGT-3′ and R: 5′-GGACTACCAGGGTATCTAATCCTGTT-3′), previously described (Nadkarni et al., 2002). *Escherichia coli* DH5-Alpha was used as control for bacteria quantification standard curve. For Firmicutes phylum quantification, sequences of primers previously described (F: 5′-GGAGYATGTGGTTTAATTCGAAGCA-3′ and R: 5′-AGCTGACGACAACCATGCAC-3′) were used (Guo et al., 2008). *Clostridium perfringens* ATCC 13124 was used as control for Firmicutes standard curve. Forward primer, 5′-GGARCATGTGGTTTAATTCGATGAT-3′ and reverse primer 5′-AGCTGACGACAACCATGCAG-3′ were used to quantify Bacteroidetes phylum bacteria, generating a 126-bp amplicon (Guo et al., 2008). *Bacteroides fragilis* ATCC 25285 was used as positive control for Bacteroidetes phylum. For *C. albicans* quantification, primers previously reported by Rahn et al. (2016) were used (FP: CCTGTTTGAGCGTCRTTT RP: TCCTCCGCTTATTGATATGC), based on the 229-bp fragment of conservative sequence of *18S rRNA* gene.

All primers were evaluated for their specificity in the software BLAST (Basic Local Alignment Search Tool - http://www.ncbi.nlm.nih. gov/blast/) and have been confirmed to be specific for their purposes. DNA samples of control strains were serially diluted by 10-fold (five dilution points; 10^1^–10^5^ ng/ml) to generate standard curve for determining the absolute target quantity in samples. More specifically, the software (Sequence Detection Software version 1.3.1, Applied Biosystems, Foster City, CA, USA) measures the amplification of the target in both the standard dilution series and test samples. A standard curve was generated using data from the standard dilution series. According to the standard curve, the software interpolates the absolute quantity of the target in the test samples.

### Statistical Approach

Data were statistically analyzed using the SPSS package for Windows, version 21.0 (SPSS, Inc., Chicago, IL, USA). Data normality was tested using the Shapiro-Wilk test and Levene test, respectively. Variables that violated the premises of analysis of variances were transformed (Samal et al., 1999) to the logarithm of 10 (total bacteria, Bacteroidetes, and Firmicutes data) and for inverse transformation (*C. albicans* data).

Descriptive statistics considered the median and interquartile range due to the non-Gaussian distribution. Within subjects’ effects were obtained using repeated measures analysis of variance (RMANOVA). Assumptions of sphericity were tested using Mauchly’s test of sphericity. Greenhouse-Geisser correction and Huynh-Feldt correction were made considering the cut-off point of 0.75 for *ϵ* values when the *p*-value of Mauchly’s test of sphericity was significant. Bonferroni adjustment of *p*-value corrected the level of significance of *α*, avoiding family-wise error.

## Results and Discussion

### Patients’ Profile Submitted to Impacted Lower Third Molar Extraction

From 2016 to 2020, 172 patients seek for third molar surgery in the private clinic at Bragança Paulista-SP where they were recruited. One hundred and thirty-three patients (77.3%) had no infection before the surgery; a minor rate of 22.7% (39 patients) showed infections, which were classified as pericoronitis (37 patients), and abscess of dental origin (two patients). In total, 288 teeth were extracted, being 195 (67.7%) impacted.

It is important to highlight that the use of antibiotic in patients with previous infection is necessary and is not the purpose of this study. While the major clinical indications for surgery in the present investigation were due to orthodontic treatment [211 teeth (73.3%)], the minor were due to carious process or coronary destruction [13 teeth (4.5%)]. From 39 patients with previous infection, 38 received amoxicillin (875 mg every 12 h) together with 0.12% chlorhexidine mouthwash, for three consecutive days before the dental surgery, followed by amoxicillin 875 mg therapy for 4 days after the dental procedure, to complete a 7-day therapy protocol. One patient needed hospital internment for the abscess treatment before the surgery procedures. This patient received amoxicillin 875 mg and 0.12% chlorhexidine mouthwash together with metronidazole (500 mg every 12 h), for 7 days. According to the literature, this protocol is necessary for anaerobic bacteria infection (Sukegawa et al., 2019; Dallaserra et al., 2020). The 133 patients without previous infection received 0.12% chlorhexidine mouthwash, for three consecutive days before the surgery; amoxicillin (875 mg, single dose), 1 h before surgery; and amoxicillin (875 mg every 12 h) for 7 days, after the surgery. The patients included in the microbiological analysis were selected from this last group, if they had impacted lower third molar. No patients had postsurgery infection in the evaluated period.

### Oral Microorganisms Quantification

**Figure 1:**
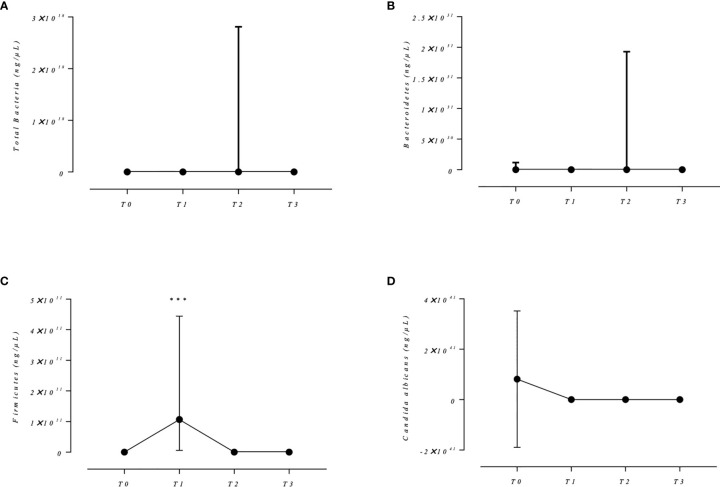
Absolute quantification of total bacteria, Bacteroidetes and Firmicutes Phyla, and *C. albicans* from oral cavity of healthy patients submitted to impacted third molar surgery, after antiseptic and antibiotic intervention. T0: before surgery; T1: after 0.12% chlorhexidine mouthwash, for 3 days; T2: after amoxicillin 875 mg, single dose, 1 h before surgery, followed by amoxicillin 875 mg every 12 h, for 7 days after surgery; T3: 28 days after amoxicillin use. Statistical analyses were performed with a sample of 11 volunteers. ηp²: Partial eta squared. Total bacteria, Bacteroidetes, and Firmicutes data were transformed by log 10 to achieve the RMANOVA premises. *Candida albicans* data were reciprocally transformed considering 1/*x*. **(A)** Median and interquartile range of total bacteria levels (ng/µl). RMANOVA statistics for total bacteria: *p*-value = 0.387, power = 0.14, ηp² = 0.100. Significant *p*-value for Mauchly’s test of sphericity required the Greenhouse-Geisser correction. **(B)** Median and interquartile range of Bacteroidetes levels (ng/µl). RMANOVA statistics for Bacteroidetes: *p*-value = 0.396, power = 0.16, ηp² = 0.119. Significant *p*-value for Mauchly’s test of sphericity required the Greenhouse-Geisser correction. **(C)** Median and interquartile range of Firmicutes levels (ng/µl). RMANOVA statistics for Firmicutes: *p*-value = 0.000, power = 1.00, ηp² = 0.949. Mauchly’s sphericity was assumed. ***Means *p*-value lower than 0.0000 after Bonferroni adjustment for multiple comparisons. **(D)** Median and interquartile range of *Candida albicans* levels (ng/µl). RMANOVA statistics for *Candida albicans*: *p*-value = 0.101, power = 0.51, ηp² = 0.203. Mauchly’s sphericity was assumed.

Total bacteria, Bacteroidetes, and *C. albicans* load did not differ across the follow-up (T0 *vs.* T1; T0 *vs.* T2, and T0 *vs.* T3) (**Table 1** and **Figures 1A, B, D**). These findings are not in accordance with the professionals’ protocols of using antiseptic or antibiotic prophylaxis to decrease the bacterial load in order to reduce the postsurgery infections (Lacasa et al., 2007; Luaces-Rey et al., 2010). Despite the fact that no significant changes have been observed in the total bacteria, there was a significant difference between the Firmicutes levels across the follow-up. This effect seems to be large (*ηp² = 0.94*). Interestingly, *post-hoc* test demonstrated that the levels of Firmicutes in T1 (chlorhexidine antiseptic usage) were higher than T0, T2, and T3 (**Figure 1C** and **Table 1**). The oral microbiota has microorganisms with diversity of energetic metabolism, including facultative anaerobic and anaerobic species. Coagulase-negative *Staphylococcus* and viridans group *Streptococcus* represent the facultative anaerobes, while *Fusobacterium* spp., *Prevotella* spp., *Porphyromonas* spp., *Bacteroides fragilis*, *Clostridium* spp., and *Treponema* spp. represent the aerobic group. Coagulase-negative *Staphylococcus*, viridans group *Streptococcus*, and *Clostridium* spp. compose the Firmicutes phylum, representing a significant part of oral potential pathogens (Robertson and Smith, 2009). Increase in the Firmicutes level may act to favor the selection of the oral pathogens in healthy individuals, since chlorhexidine antiseptic-based mouthwashes are sold without a prescription and, a previous study in our laboratory showed that they are daily used by healthy individuals after tooth brushing (Russi et al., 2020).

**Table 1:** Within subjects’ effects of antiseptic and antibiotic intervention across the time in total bacteria, Bacteroidetes and Firmicutes Phyla, and *C. albicans*: a repeated measures analysis of variance.

Interventions across the time	Total bacteria	Bacteroidetes	Firmicutes	*Candida albicans*
	Median (IQR)	Median (IQR)	Median (IQR)	Median (IQR)
***T0***	6.27 × 10^3^ (4.04 × 10^10^)	9.07 × 10^6^ (1.16 × 10^28^)	1.82 × 10^2^ (8.13 × 10^3^)	8.15 × 10^38^ (2.70 × 10^39^)
***T1***	0.175 × 10^2^ (8.25 × 10^7^)	2.01 × 10^6^ (2.92 × 10^20^)	1.06 × 10^11^ (4.38 × 10^9^)***	5.64 × 10^16^ (6.13 × 10^12^)
***T2***	1.93 × 10^4^ (2.81 × 10^16^)	4.94 × 10^23^ (1.93 × 10^29^)	5.44 × 10^3^ (5.66 × 10^3^)	6.57 × 10^8^ (2.18 × 10^9^)
***T3***	1.30 × 10^3^ (1.14 × 10^10^)	5.24 × 10^16^ (2.30 × 10^26^)	4.58 × 10^3^ (8.02 × 10^3^)	1.67 × 10^14^ (5.55 × 10^14^)
**RMANOVA**				
***p*-Value (power)**	0.387 (0.14) ***β***	0.396 (0.16) ***β***	0.000 (1.00)	0.101 (0.51)
***ηp²***	0.100	0.119	0.949	0.203

Statistical analyses were performed with a sample of 11 volunteers. ηp², partial eta squared. Total bacteria, Bacteroidetes, and Firmicutes data were transformed by log 10 to achieve the RMANOVA premises. C. albicans data were reciprocally transformed considering 1/x.

**β**, significant p-value for Mauchly’s test of sphericity required the Greenhouse-Geisser correction.

***Means p-value lower than 0.0000 after Bonferroni adjustment for multiple comparisons.

The statistical significance observed only for the Firmicutes phylum load after antiseptic usage may be the result of microbial dysbiosis caused by antiseptic therapy. The dysbiotic condition of the microbiota is associated with systemic disorders (Plachokova et al., 2021), and it may induce the selection of antibiotic-resistant strains, which can result in a therapeutic failure in a future infection episode (Khalil et al., 2016; Neves et al., 2020). According to Khalil et al. (2016), a single dose of amoxicillin is sufficient to cause an ecological disturbance in the oral microbiota and induce selection of resistant strains. In addition, studies have been showing a cross-resistance to antibiotics after antiseptic usage (Beier et al., 2021; Garratt et al., 2021; Merchel Piovesan Pereira et al., 2021). Furthermore, even after 28 days after prophylaxis, the oral bacteria quantification did not seem to return to baseline levels (T0 *vs.* T3), suggesting that the patients may not have been able to reestablish their microbiota after the dysbiosis (**Table 1**). This is a preliminary study using a convenience sample. The major problem of a convenience sample is the selection bias. To avoid that, only healthy patients, without previous infection and comorbidities, of a single dental office, operated by the same dentist were included. Furthermore, all patients received from researchers, the same brand of antiseptic and antibiotic for prophylaxis. In addition, the power of the test and the effect sizes were included. Noticeably, the statistical difference among the experimental phases regarding Firmicutes phylum level has a large power (1.00) and a large effect size (0.949) (**Table 1**).

Finally, our results showed that most patients seeking for third molar surgery had no presurgical infections, and even so received antiseptic and antibiotic prophylactic therapies. If no antibiotics were used for this group of patients, we could certainly contribute to a decrease in the resistance rates, as well as to diminish therapeutic failure in the treatment of infectious diseases (WORLD HEALTH ORGANIZATION, 2015). Investment in biosafety during the dental surgical procedures must be used as a tool to reduce antibiotics prescription to prevent infections.

## Conclusion

The discussion about antibiotic usage in dentistry is not recent, and because of the lack of consensus, there is no safety protocol to avoid dysbiosis and antibiotic resistance. Further well-designed studies involving microbiome sequencing are necessary to determine what species are suitable to selection during antiseptic and antibiotic therapy. In spite of not identifying these species, our preliminary results warn about the need of personalized tools and protocols in dentistry, targeting individual particular requirements, avoiding the use of a generalized predetermined protocol.

## Prior Presentation

Partial results of this study were presented at the 31th ECCMID, Vienna, Austria, 2021.

## Data Availability Statement

The original contributions presented in the study are included in the article/supplementary material. Further inquiries can be directed to the corresponding author.

## Ethics Statement

The studies involving human participants were reviewed and approved by Universidade São Francisco. The patients/participants provided their written informed consent to participate in this study.

## Author Contributions

CN and CA contributed with study design, conduction of experiments, data analysis, and writing of the manuscript. NR, KFR, KLR, and AP contributed for conduction of experiments. TP contributed with data analysis and writing and revision of the manuscript. RG contributed with study design, conduction of experiments, data analysis, and writing and revision of the manuscript. All authors contributed to the article and approved the submitted version.

## Conflict of Interest

The authors declare that the research was conducted in the absence of any commercial or financial relationships that could be construed as a potential conflict of interest.

## Publisher’s Note

All claims expressed in this article are solely those of the authors and do not necessarily represent those of their affiliated organizations, or those of the publisher, the editors and the reviewers. Any product that may be evaluated in this article, or claim that may be made by its manufacturer, is not guaranteed or endorsed by the publisher.
